# Frequency and phenotype of headache in covid-19: a study of 2194 patients

**DOI:** 10.1038/s41598-021-94220-6

**Published:** 2021-07-19

**Authors:** David García-Azorín, Álvaro Sierra, Javier Trigo, Ana Alberdi, María Blanco, Ismael Calcerrada, Ana Cornejo, Miguel Cubero, Ana Gil, Cristina García-Iglesias, Ana Guiomar Lozano, Cristina Martínez Badillo, Carol Montilla, Marta Mora, Gabriela Núñez, Marina Paniagua, Carolina Pérez, María Rojas, Marta Ruiz, Leticia Sierra, María Luisa Hurtado, Ángel Luis Guerrero Peral

**Affiliations:** 1 Headache Unit, Department of Neurology, Hospital Clínico Universitario de Valladolid, Avenida Ramón y Cajal N° 3, 47003 Valladolid, Spain; 2 Valladolid East Primary Care Basic Health Area, Valladolid, Spain; 3 Department of Medicine and toxicology, University of Valladolid, Valladolid, Spain

**Keywords:** Microbiology, Neuroscience, Health care, Medical research, Neurology

## Abstract

To estimate the frequency of headache in patients with confirmed COVID-19 and characterize the phenotype of headache attributed to COVID-19, comparing patients depending on the need of hospitalization and sex, an observational study was done. We systematically screened all eligible patients from a reference population of 261,431 between March 8 (first case) and April 11, 2020. A physician administered a survey assessing demographic and clinical data and the phenotype of the headache. During the study period, 2194 patients out of the population at risk were diagnosed with COVID-19. Headache was described by 514/2194 patients (23.4%, 95% CI 21.7–25.3%), including 383/1614 (23.7%) outpatients and 131/580 (22.6%) inpatients. The headache phenotype was studied in detail in 458 patients (mean age, 51 years; 72% female; prior history of headache, 49%). Headache was the most frequent first symptom of COVID-19. Median headache onset was within 24 h, median duration was 7 days and persisted after 1 month in 13% of patients. Pain was bilateral (80%), predominantly frontal (71%), with pressing quality (75%), of severe intensity. Systemic symptoms were present in 98% of patients. Headache frequency and phenotype was similar in patients with and without need for hospitalization and when comparing male and female patients, being more intense in females.

Trial registration: This study was supported by the Institute of Health Carlos III (ISCIII), code 07.04.467804.74011 and Regional Health Administration, Gerencia Regional de Salud, Castilla y Leon (GRS: 2289/A/2020).

## Introduction

Headache is one of the most frequent non-respiratory symptoms of coronavirus disease 2019 (COVID-19)^1,2^. There is a significant disparity in the reported prevalence, being described by 14–70% of COVID-19 patients depending on the study^1–5^. According to the Centers for Disease Control and Prevention (CDC), it is the most frequent neurological symptom, experienced by 14.8% of hospitalized patients, and reaches a frequency of 22.7% of patients between 18 and 49 years old^6^. The true frequency and phenotype of headache with COVID-19 is still unclear, as most of the available studies are series of hospitalized patients^1–6^.

The first series in confirmed COVID-19 patients described headache presentation within the first 72 h of the disease in most cases^7–9^. The phenotype exhibits a bilateral headache with frontal predominance, an oppressive quality, and moderate to severe intensity^8^. Most patients fulfill the International Classification of Headache Disorders (ICHD) criteria for “Acute headache attributed to systemic viral infection”; however, 54% of patients also fulfilled the phenotypic ICHD criteria for tension-type headache (TTH) and 25% for migraine^7,10^. Despite the fact that the phenotype could be a chameleon of a primary headache, all cases in hospital-based series exhibited at least one red flag^9^. It is unknown whether headache can be misdiagnosed as a primary headache disorder in COVID-19 patients managed in an outpatient setting. In hospitalized patients, the presence of headache independently predicted a lower risk of mortality^2^, and lower risk of intensive care-unit admission^11,12^. However, patients with headache also described a high degree of disability, and need for acute treatment was frequent^7^. The aims of this study are to estimate the incidence of headache over the COVID-19 disease course in the general population and to characterize the clinical phenotype of headache in patients with confirmed COVID-19. We compared both frequency and clinical phenotype in patients that were hospitalized with those managed in an outpatient basis, and we compared both sexes as well.

## Methods

### Study design and setting

This is an observational analytic study with a cross-sectional design. We followed the Strengthening in the Reporting of Observational Studies in Epidemiology (STROBE) statement^13^. The study population included patients with confirmed COVID-19 and presence of headache at any point over the COVID-19 disease course. The study was done in the Valladolid East Healthcare Area, which includes a tertiary academic hospital (Hospital Clínico Universitario de Valladolid) and 22 primary care centers. The reference population was 261,431 patients in total, with the reference population of the included primary care centers ranging between 1896 and 20,930 patients, including both rural areas and urban centers^12^.

### Study population

The eligibility criteria was based on prior studies^2,7,9^; patients were included if: (1) they had a confirmed diagnosis of COVID-19, either by real-time reverse-transcription polymerase chain reaction (RT-PCR) assay from a respiratory tract sample and/or by the presence of anti-severe acute respiratory syndrome coronavirus 2 (SARS-CoV-2) antibodies; (2) new-onset headache presented during the course of COVID-19 that was not better accounted for by another secondary cause, according to the International Classification of Headache Disorders, 3rd edition (ICHD-3)^10^ criteria; and (3) they were older than 18 years. Patients were excluded if: (1) they were not able to participate due to a severe or unstable medical condition, (2) they had prior history of cognitive impairment or dementia, (3) they had speech or language disturbances that made them unable to participate in the evaluation; (4) they expired during the course of COVID-19, (5) or if they declined to participate. We also excluded those patients who we were unable to reach after at least three attempts using all available contact options.

### Screening of patients

According to the local protocol, patients with suspected COVID-19 symptoms were asked to call a 24-h COVID-19 hotline specifically created for the pandemic. Patients who reported symptoms suggestive of COVID-19 were followed daily or every other day by phone or in-person. Headache was included on the COVID-19 symptoms checklist. The Emergency Department protocol also included a standardized checklist with headache as a symptom to evaluate (supplementary Fig. 1). Criteria used for hospital admission, according to the local and national protocols^14^, is available in supplementary materials. We identified patients from the database of primary care COVID-19 teams and hospital registrations and admissions. We systematically screened all patients that were evaluated between March 8, 2020 (first confirmed COVID-19 case in the healthcare area) and April 11, 2020. The presence of headache was obtained from electronic health records. In addition, in addition, all covid-19 patients were interviewed directly about the presence of headache by a member of the study team, which was composed of two neurologists and 16 primary care physicians with prior training in headache disorders. Patients were scheduled for in-person evaluations when possible or were evaluated over the phone. All patients were evaluated at least 1 month after the onset of symptoms to ensure that late-onset headache would be detected. Patients were asked about the presence of headache and were invited to complete the full questionnaire.

### Study objectives

The main objectives were: (1) to estimate the incidence of headache over the course of COVID-19 in the general population and (2) to characterize the clinical phenotype of the headache in patients with COVID-19.

The secondary objectives were: (1) to evaluate the frequency and types of red flags in patients with a headache, (2) to compare the clinical phenotype of headache between patients who needed hospitalization to those who did not; (3) to evaluate the clinical phenotype depending on the sex of the patients.

## Variables

All researchers were trained prior to the study onset. The study questionnaire was standardized and based on prior studies^7,15^. A study coordinator monitored adherence to the protocol and the accuracy and completeness of the data.

Demographic data included age, sex, and country of origin. A physician on the research team assessed prior medical history and family history of migraine. Prior medical history was evaluated for the presence of hypertension, diabetes, a smoking habit, cardiac disorders, pulmonary disorders, cancer, immunosuppression, and chronic neurologic disorders (full definition available in supplementary material 1). A physician also analyzed prior history of headache, including the specific diagnosis, the source of the diagnosis, the frequency of headache (days per month in the preceding 3 months), and the degree of similarity between the headache experienced during the course of COVID-19 and their usual headache on a 0–100% rating scale (0: completely different, 100%: exactly the same). Finally, a physician also asked all participants about the prior history of headache in temporal relation to prior infections and whether those were similar to the present one.

Clinical presentation of COVID-19 included radiological abnormalities, and the need for oxygen therapy was determined. We analyzed whether the COVID-19 diagnosis was based on RT-PCR and/or serological tests. We assessed the first symptom and the occurrence of other systemic symptoms (full list in supplementary materials). We evaluated the time elapsed between the first symptom and the headache onset. Headache phenotype variables included laterality (bilateral, unilateral or midline headache), topography, quality of pain, presence of associated symptoms (photophobia, phonophobia, osmophobia, nausea, vomiting, cranial autonomic symptoms (any of the following: conjunctival injection, lacrimation, nasal congestion, rhinorrhea, eyelid edema, forehead or facial sweating, ptosis), avoidance of routine physical activity), intensity of pain (rated on a 0–10 numeric rating scale, 0: no pain, 10: worst possible pain), degree of disability caused by the headache (rated on a 0–100% numeric rating scale, 0%: no disability, 100%: absolute disability), need for and type of acute medication, and factors that worsened the headache (walking, head movements, ocular movements, coughing, bending or sneezing). Finally, we screened for the presence of headache-related red flags, according to a standardized and previously used in COVID-19 patients with headache^9^ based on the SNNOOP10 list^16^ (supplementary appendix).

### Statistical analysis

We present qualitative and ordinal variables as frequencies and percentages. Variables with missing data are presented as the proportion of patients exhibiting the variable over the total number of patients with valid data. We describe continuous variables as means and standard deviations (SD) or medians and interquartile ranges (IQR) depending on the distribution. Normality of the distribution was analyzed by the Kolmogorov–Smirnov test. Incidence of headache was calculated as cases per hundred patients (%) with the 95% confidence interval (CI). Data were analyzed both per intention to treat (ITT), dividing the number of COVID-19 cases presenting with headache by the total number of confirmed COVID-19 cases (i.e. entire population of the area, including those that were not able to be screened for the study); and per protocol (PP), dividing the number of COVID-19 cases presenting with headache by the population that we were able to screen. Qualitative categorical variables were compared using Fisher’s exact tests. Quantitative variables were assessed by Student’s t-tests if the distribution was normal and the variance was homogeneous or with the Mann–Whitney U test in the rest of the cases. The sample size was not estimated in advance and the analysis was completed using the available data. Missing data were managed with complete case analysis. The significance level was set to *P* ≤ 0.05 and was corrected in the case of multiple comparisons with the False Discovery Rate (FDR) using the Benjamini–Hochberg procedure^17^. The statistical analysis was done with SPSS statistical software package (version 26.0) for Mac (IBM Corp. Armonk, NY).

### Ethical aspects

The study was done in accordance with the International Conference on Harmonization Guidelines for Good Clinical Practice and the Declaration of Helsinki principles. Valladolid East ethics review board approved both the hospital series (PI 20-1738) and the primary care series (PI 20-1881). All participants gave written informed consent.

### Ethics approval and consent to participate

The local ethics review board approved both the hospital series (PI 20-1738) and the primary care series (PI 20-1881). All participants gave verbal or written informed consent.

### Consent for publication

Figures were created with BioRender.com by the corresponding author.

### Conference presentation

The present study was partially presented at the Migraine Trust International Symposium 2020.

## Results

### Frequency of headache in COVID-19

**Figure 1:**
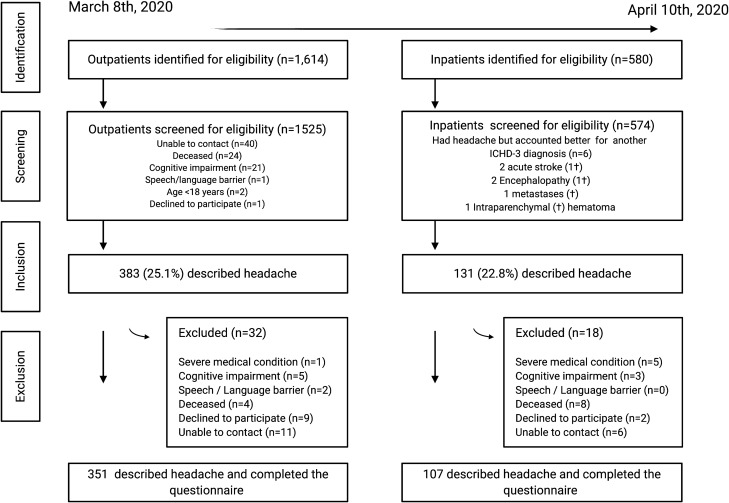
Flow diagram. Number of patients that were identified, screened, included, and excluded, with the specific reasons for non-participation.

**Figure 2:**
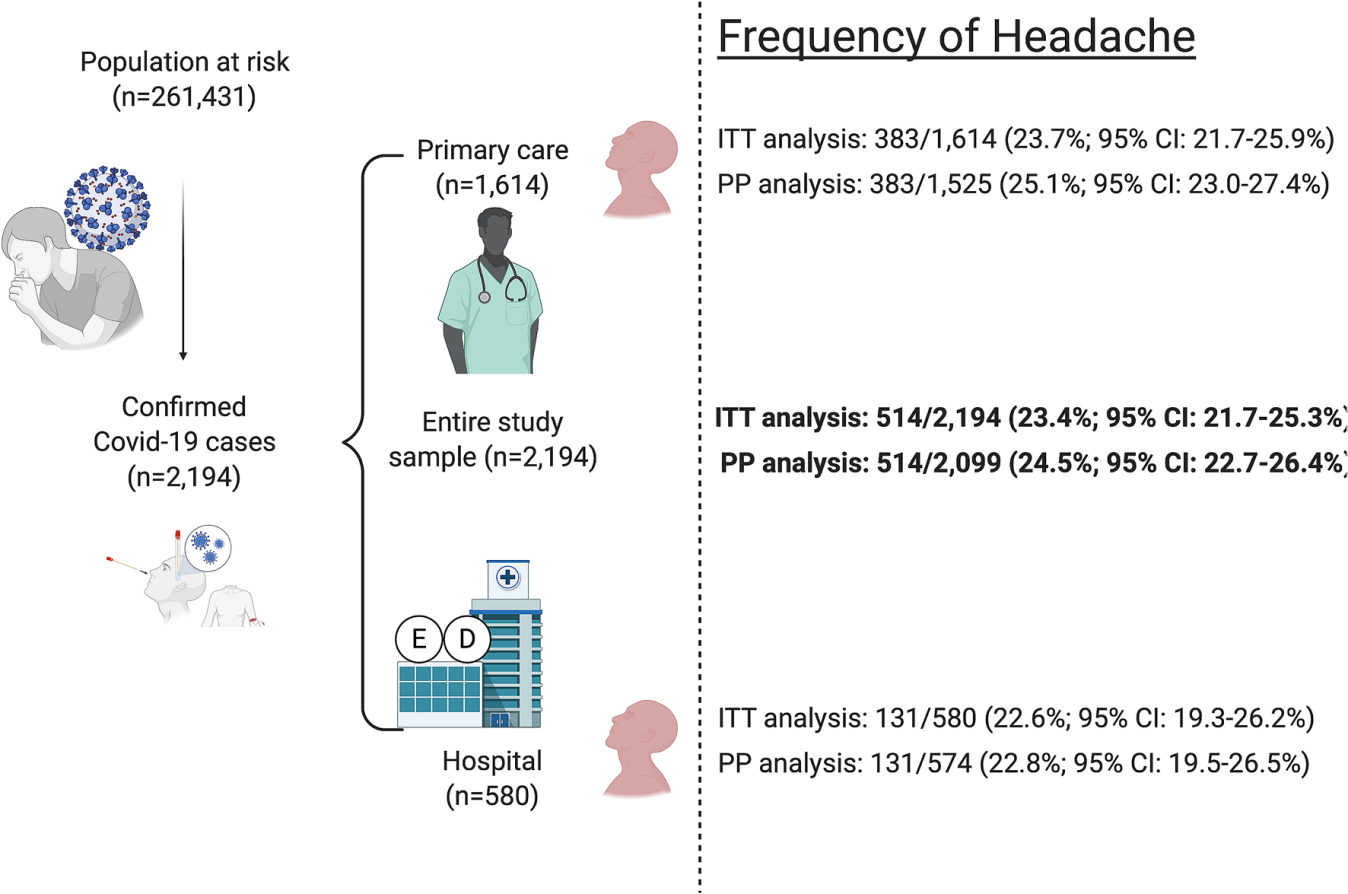
Frequency of headache in the entire sample and in the groups of patients managed in primary care and hospital care settings. *ITT* Intention-to-treat. *PP* Per protocol.

During the study period, 2194 patients out of the at-risk population of 261,431 were diagnosed with COVID-19, leading to a COVID-19 incidence of 8.4 cases per 1000 people. Figure 1 represents the flow diagram of identified, screened, included and excluded patients. The frequency of headache in the entire study sample and in the sub-groups of outpatient and hospitalized patients is presented in Fig. 2. In total, 458 out of 514 (89.1%) participants completed the questionnaire and were included in the analysis of headache phenotype.

Number of patients that were identified, screened, included, and excluded, with the specific reasons for non-participation.

### Demographic data

The age of headache patients ranged between 18 and 97 years, with a median age of 51 (IQR: 42–61) years. Regarding sex, 320 (72.1%) patients were female. Patients were born in Spain in 416 (90.8%) cases, in Latin-American countries in 30 (6.6%), in other European countries in four (0.9%), in North-African countries in two (0.4%), and in Sub-Saharan Africa in one (0.2%). Country of origin was not specified in five (1.1%) cases.

### Prior medical history

Patients had a prior history of hypertension in 98 (21.4%) cases, pulmonary disorders in 53 (11.6%), cardiac disorders in 49 (10.7%), diabetes in 37 (8.1%), prior history of cancer in 31 (6.8%), smoking habit in 27 (5.9%), chronic neurologic disorders in 12 (2.6%), and immunosuppression in 11 (2.4%).

### Prior history of headache

Family history of migraine was reported by 117 (25.5%) patients. Prior history of headache was described by 223 (48.7%) patients, with migraine in 83 (18.1%), TTH in 99 (21.6%), or others. Detailed information about diagnosis and the source of the diagnosis is available in supplementary materials. Median frequency of the prior headache was 1 day per month (IQR 0–2). Patients judged the similarity between the present headache and the usual headache as 0% (median; IQR 0–50%) similar. Patients described prior episodes of headache in temporal relation to prior infections in 159 (34.7%) cases and described the headache as similar to the present episode in 99 (62.2%) of those 159 cases.

### COVID-19 presentation and diagnosis

Systemic symptoms were present in 439/450 (97.6%) patients, and systemic symptoms and/or anosmia or myalgia were present in 443/450 (98.4%) patients. Asthenia, cough, anosmia and fever were the most frequent concomitant symptoms (Supplementary table 1). Information about the most bothersome symptom was available in 435 cases, described as headache in 68 (15.6%), asthenia in 54 (12.4%), fever in 49 (11.3%), cough in 44 (10.1%) and diarrhea in 26 (6.0%). COVID-19 diagnosis was based on PCR in 455 (99.3%) cases and serology in 88 (19.2%) cases. Pneumonia was present in 152 (33.2%) cases, and 86/447 (19.2%) patients needed oxygen therapy.

### Headache over the course of the COVID-19 disease

Headache was the most frequent first symptom of COVID-19, described by 128 (27.9%) of the patients who reported headache, followed by fever in 109 (23.1%), cough in 60 (13.1%), asthenia in 32 (7.0%). Headache was the first COVID-19 symptom in at least 128/2194 (5.8%) patients when COVID-19 patients without headache were included. The median time from the first COVID-19 symptom to headache was 1 day (IQR 0–3, n = 428). Headache started within the first day of symptoms in 174/428 (40.7%) patients, on the second day in 52 (12.1%), on the third day in 61 (14.3%), on the fourth day in 42 (9.8%) patients and on the fifth day or later in 99 (23.1%) patients. Headache persisted longer than 1 month of after the resolution of the general COVID-19 symptoms in 59 (12.9%) patients. In the rest of the sample, the median duration of the headache was 7 days (IQR: 4–14, n = 374).

### Phenotype of headache

Table 1 summarizes the main phenotypic variables of headache. The headache was holocranial in 81/433 (18.7%) patients. Median daily duration of headache was seven hours (IQR 3–24, n = 390). The headache lasted between 1 and 6 h in 191/390 (49.0%) patients, between 7 and 12 h in 57/390 (14.6%), between 13 and 18 h in 14/390 (3.6%), and between 19 and 24 h in 128/390 (32.8%). Median intensity of the headache was 7 out of 10 (IQR 6–8), and patients judged the degree of disability caused by the headache as 50% (IQR 20–80%). Symptomatic medication was needed by 413/437 (94.5%) patients, including acetaminophen in 382/413 (92.5%) cases, ibuprofen in 71/413 (17.2%) cases, and metamizole in 51/413 (12.3%) cases. The full list of acute medications is available in supplementary materials.

**Table 1:** Phenotype of the headache.

Characteristic	Frequency (n, %)	Sample (n)
*Laterality*		
Bilateral	350 (80.1%)	437
Unilateral	84 (19.2%)	437
Midline	3 (0.7%)	437
*Topography of pain*		
Frontal	312 (70.9%)	433
Temporal	190 (43.9%)	433
Parietal	136 (31.4%)	433
Occipital	141 (32.6%)	433
Periocular	170 (39.3%)	433
Vertex	113 (26.2%)	431
Cervical	118 (27.3%)	433
*Quality of pain*		
Pressing	324 (74.7%)	434
Throbbing	64 (14.8%)	433
Stabbing	68 (15.7%)	434
Electric	4 (0.9%)	434
Burning	9 (2.1%)	434
*Associated symptoms*		
Photophobia	152 (33.3%)	456
Phonophobia	147 (32.3%)	456
Osmophobia	16 (3.7%)	431
Avoidance of physical activity	289 (66.9%)	432
Nausea	70 (16.4%)	428
Vomiting	21 (4.6%)	457
Cranial autonomic symptoms	83 (24.8%)	335
Worsening with physical activity	52 (11.4%)	458
Worsening by with head movements	135 (31.2%)	433
Worsening with ocular movements	79 (18.2%)	457

Red flags regarding the headache phenotype were present in 264/432 (61.1%) of patients, with the most frequently described being the “worst headache ever experienced” in 26% of patients (Table 2).

**Table 2:** Frequency of red flags.

Red flag	Frequency (n, %)	Sample (n)
Fever	263 (59.2%)	444
Worst headache ever experienced	113 (26.0%)	343
Wake-up headache	97 (21.2%)	458
Precipitated by coughing	93 (21.5%)	433
Treatment resistant	84 (19.4%)	434
Precipitated or aggravated after sitting upright or standing	63 (14.5%)	434
Progressive headache	60 (13.1%)	458
Precipitated by bending	59 (12.9%)	458
Confusion	27 (6.2%)	434
Sudden onset	25 (5.8%)	434
Precipitated after lying horizontally	24 (5.5%)	434
Loss of consciousness	2 (0.5%)	433

### Inpatient vs. outpatient comparison

Patients who needed hospitalization were older and more frequently had cough, had pneumonia, and needed oxygen therapy; outpatient cases more frequently had asthenia, fever, and weakness and described temporal topography of the headache and photophobia more frequently (Table 3).

**Table 3:** Comparison of demographic variables, frequency of associated symptoms and main phenotypic variables of the headache between inpatient and outpatient cases.

Variable	Entire study sample (n = 458)	Inpatient cases (n = 107)	Outpatient cases (n = 351)	FDR Adjusted *P* value
Median age (years)	51 (42–61)	56 (IQR 48–65)	50 (IQR 40–60)	**0.002**
Female sex	330/458 (72.1%)	69/107 (64.5%)	261/351 (74.4%)	0.100
Prior history of headache	223/449 (49.7%)	60/107 (56.1%)	163/342 (47.7%)	0.195
Headache as the first symptom	128/446 (28.7%)	32/107 (29.9%)	96/339 (28.3%)	0.911
Duration of headache (days)	7 (IQR 4–14)	7 (IQR 4–10)	7 (IQR 4–15)	0.098
Anosmia	269/448 (60%)	69/107 (64.5%)	200/341 (58.7%)	0.366
Asthenia	337/458 (73.6%)	55/107 (51.4%)	282/351 (80.3%)	**0.001**
Cough	294/446 (65.9%)	87/107 (81.3%)	207/339 (61.1%)	** < 0.001**
Diarrhea	185/447 (41.4%)	51/107 (47.7%)	134/340 (39.4%)	0.197
Dyspnea	179/444 (40.3%)	52/107 (48.6%)	127/337 (37.7%)	0.094
Fever	263/444 (59.2%)	47/107 (43.9%)	216/337 (64.1%)	** < 0.001**
Myalgia	228/443 (51.5%)	50/107 (46.7%)	178/336 (53.0%)	0.333
Weakness	238/446 (53.4%)	27/107 (25.2%)	211/339 (62.2%)	** < 0.001**
Presence of pneumonia	152/450 (33.8%)	102/107 (95.3%)	50/343 (14.6%)	** < 0.001**
Need for oxygen	86/447 (19.2%)	55/107 (51.4%)	31/340 (9.1%)	** < 0.001**
Headache as the most bothersome symptom	68/428 (15.9%)	21/100 (21.0%)	47/328 (14.3%)	0.172
Median intensity (0–10 NRS)	7 (IQR 6–8)	7 (IQR 6–8)	7 (IQR 6–8)	0.083
Median impairment (0–100% NRS)	50% (IQR 20–80%)	60 (IQR 40–80)	50 (IQR 20–80)	0.151
Frontal topography	307/433 (70.9%)	84/107 (78.5%)	223/326 (68.4%)	0.093
Temporal topography	190/433 (43.9%)	34/107 (31.8%)	156/326 (47.9%)	**0.011**
Pressing quality	324/434 (74.7%)	80/107 (74.8%)	244/327 (74.6%)	> 0.999
Photophobia	152/456 (33.3%)	48/105 (45.7%)	104/351 (29.6%)	**0.009**
Phonophobia	147/456 (32.2%)	41/105 (39.0%)	106/351 (30.2%)	0.147
Avoidance of physical activity	289/432 (66.9%)	62/107 (57.9%)	227/325 (69.8%)	0.065
Worsening with physical activity	135/433 (31.2%)	33/107 (30.8%)	102/326 (31.3%)	> 0.999
Need for acute medication	413/437 (94.5%)	101/107 (94.4%)	312/330 (94.5%)	> 0.999

Significance level was adjusted after multiple comparisons with the False Discovery Rate (FDR) using the Benjamini–Hochberg procedure. In bold, statistically significant results. *NRS* Numeric Rating Scale.

### Sex differences

When comparing female and male patients, female patients had a prior history of headache more frequently. Regarding the headache, female patients described a higher intensity and described phonophobia more frequently (Table 4).

**Table 4:** Comparison of demographic variables, frequency of associated symptoms and main phenotypic variables of the headache between males and females.

Variable	Entire study sample (n = 458)	Males (n = 128)	Females (n = 330)	FDR adjusted *P* value
Median age (years)	51 (42–61)	48 (40–60)	53 (43–62)	0.05
Prior history of headache	223/449 (49.7%)	50/128 (39.1%)	173/321 (53.9%)	**0.042**
Headache as the first symptom	128/446 (28.7%)	28/127 (22.0%)	100/319 (31.3%)	0.225
Duration of headache (days)	7 (IQR 4–14)	7 (IQR 4–10)	8 (IQR 4–16)	0.227
Anosmia	269/448 (60%)	81/128 (63.3%)	188/320 (58.8%)	0.705
Asthenia	337/458 (73.6%)	96/128 (75.0%)	241/330 (73.0%)	0.862
Cough	294/446 (65.9%)	93/128 (72.7%)	201/318 (63.2%)	0.254
Diarrhea	185/447 (41.4%)	54/128 (42.2%)	131/319 (41.1%)	0.905
Dyspnea	179/444 (40.3%)	53/127 (41.7%)	126/317 (39.7%)	0.850
Fever	263/444 (59.2%)	80/128 (62.5%)	183/316 (57.9%)	0.760
Myalgia	228/443 (51.5%)	67/128 (52.3%)	161/315 (51.1%)	0.905
Weakness	238/446 (53.4%)	72/128 (56.3%)	166/318 (52.2%)	0.644
Presence of pneumonia	152/450 (33.8%)	50/127 (39.4%)	102/323 (31.6%)	0.254
Need for oxygen	86/447 (19.2%)	31/126 (24.6%)	55/321 (17.1%)	0.231
Headache as the most bothersome symptom	68/428 (15.9%)	22/123 (17.9%)	46/305 (15.1%)	0.616
Median intensity (0–10 NRS)	7 (IQR 6–8)	6 (IQR 5–8)	7 (IQR 6–8)	**0.002**
Median impairment (0–100% NRS)	50% (IQR 20–80%)	50 (IQR 10–70)	55 (IQR 20–80)	0.055
Frontal topography	307/433 (70.9%)	87/128 (68.0%)	220/305 (72.1%)	0.652
Temporal topography	190/433 (43.9%)	52/128 (40.6%)	128/305 (45.2%)	0.662
Pressing quality	324/434 (74.7%)	93/128 (72.7%)	231/306 (75.5%)	0.684
Photophobia	152/456 (33.3%)	34/127 (26.8%)	118/329 (35.9%)	0.237
Phonophobia	147/456 (32.2%)	29/127 (22.8%)	118/329 (35.9%)	**0.044**
Avoidance of physical activity	289/432 (66.9%)	78/128 (60.9%)	211/304 (69.4%)	0.214
Worsening with physical activity	135/433 (31.2%)	36/128 (28.1%)	99/305 (32.5%)	0.626
Need for acute medication	413/437 (94.5%)	121/128 (94.5%)	292/309 (94.5%)	> 0.999

Significance level was adjusted after multiple comparisons with the False Discovery Rate (FDR) using the Benjamini–Hochberg procedure. In bold, statistically significant results. *NRS* Numeric Rating Scale.

## Discussion

In this study, headache occurred in one of every four confirmed COVID-19 cases. Headache is a common symptom in systemic viral infections. In addition, in our sample, we estimated that headache was the first symptom of COVID-19 in 6% of patients, with an early onset in most of the cases. The phenotype of headache attributed to acute SARS-CoV-2 infection shows a bilateral headache predominantly affecting the forehead, with pressing quality, with severe intensity, and frequently accompanied by typical migraine symptoms, in line with previously published studies^7,8^. Clinicians should not misdiagnose this headache as a primary headache disorder as most cases seen in this study describe red flags^10^. The presence of systemic symptoms and the high frequency of headache-related red flags should alert to a possible secondary cause^9^.

To date, there are no known pathognomonic COVID-19 symptoms yet^18^. The most specific symptom may be anosmia^1^. The demographic profile of patients experiencing anosmia is similar to the profile of patients with headache^1,2^. However, in our series, we observed that anosmia was independently associated with headache^2,7^. The spike protein of SARS-CoV-2 binds to the angiotensin-converting enzyme 2 receptors, which are highly expressed in the respiratory epithelium^19,20^ and could explain the frontal predominance of the headache and the frequent association with anosmia and cranial autonomic symptoms.

The presence of headache might be associated with the immune response to SARS-CoV-2^2,21,22^. The early onset of the headache within the course of the disease, and the association with other symptoms such as fever, myalgia, asthenia and arthralgia, support this hypothesis^23^. Cytokine release and macrophage and lymphocyte activation have been associated with respiratory symptoms such as dyspnea and cough^24,25^. Some features of headache, such as worsening with head movement, ocular movement, photophobia or phonophobia could be caused by the above immune responses^21,22^; however, these features were not described by all patients in this study.

We attempted to ascertain whether the headache was different in patients who required hospitalization compared with the rest of the sample. As expected, hospitalized patients were older and more often had pneumonia and needed oxygen therapy. However, we did not observe differences regarding the headache phenotype or frequency, except for a slightly higher frequency of photophobia and temporal location of the headache. Interestingly, we observed that some of the general symptoms, such as asthenia, fever and weakness, were more frequent in outpatients, which may support the hypothesis that the headache is a consequence of an efficient immune response^2,26^.

The proposed reasons for the sex differences in COVID-19 prognosis are related to the immune response^27^. Prior large studies described a higher frequency of headache in female patients^2^. The present study was not designed to analyze which variables were associated with the presence of headache. However, we compared the clinical phenotype between sexes, and the only difference that we observed was a higher median intensity and a higher frequency of phonophobia in females. This could be related with a higher proportion of migraine patients in the female group, where prevalence of migraine is three times higher than in men in middle-aged women. The higher frequency of prior history of headache, the higher intensity and the increased frequency of photophobia could suggest this interpretation. The absence of other statistically significant differences should be interpreted cautiously, as this was not the primary endpoint of the study and the study might be underpowered (i.e., a possible type II error)^28^.

Headache is a disabling symptom of COVID-19^7,8^. In our sample, headache was judged by the patient to be the most bothersome symptom when compared with the rest of general and neurologic symptoms. Patients described a moderate intensity of pain, a frequent need for acute medication, and a median degree of disability of 50%. For these reasons, adequate evaluation and symptomatic treatment should be offered to patients with headache^29^. Nevertheless, use of some typical acute medications, including non-steroidal anti-inflammatory drugs, were discouraged during the pandemic, and care for headache patients was significantly less accessible as COVID-19 patients were prioritized^30–32^.

The reason why headache persists in some patients is unclear. Even considering that TTH and migraine are the second and third most prevalent diseases worldwide, respectively, the incidence of headache during the short study period exceeded the expected rate. SARS-CoV-2 infection may trigger the activation of the trigeminovascular system and meningeal nociceptors^7,8^. Future studies should analyze the presence of localized subclinical meningeal or nasal/sinus inflammation and the role of the persistently high laboratory parameters exhibited by some patients.

Our study has relevant limitations. The frequency of headache might be underestimated due to recall bias, however, the frequency observed in our study is similar to that of CDC reports^6^ and the literature^1–3,7–9^. The lack of prospective evaluation may have failed to detect those patients with multiple headaches or a changing headache phenotype over the course of the disease and recall bias could also affect the headache description. The high number of researchers involved in the study could have caused interviewer variability; however, all researchers were consistently trained and monitored during the study. The proportion of missing data was below 5%, and we managed missing data by using complete case analysis to decrease variability. In contrast, our study is not expected to be influenced by selection bias, as we screened both hospitalized and outpatient cases. Survival bias may exist, however, the percentage of patients with headache who expired was relatively low when compared with the rest of the patients. The sample size was relatively low and therefore the study might be underpowered to detect some associations. The study of patients with phenotypes similar to new daily persistent headache, and those factors associated with a longer headache duration, are still understudied. Future studies should employ headache-specific questionnaires to evaluate the disability caused by headache in a more standardized way.

## Conclusion

Headache is a frequent symptom of COVID-19. It may be the presenting symptom and typically develops early in the course of the disease. Most patients describe a bilateral headache with frontal predominance, an oppressive quality, and severe intensity. Asthenia, fever and weakness were more frequent in outpatients compared with inpatients. We did not observe overall phenotypic differences in headache between inpatients and outpatients or between sexes except for photophobia, which was more frequent in hospitalized patients and females. Prior history of headache was most frequent in female patients, who had more intense headaches during COVID-19.

## Supplementary Information

Supplementary Information.

## Data Availability

Datasheets are available upon reasonable request to the corresponding author.

## Abbreviations

COVID-19: Coronavirus disease 2019
CDC: Centers for Disease Control and Prevention
ICHD: International Classification of Headache Disorders
TTH: Tension-type headache
STROBE: Strengthening in the reporting of observational studies in epidemiology
RT-PCR: Real-time polymerase chain reaction
SARS-CoV-2: Severe acute respiratory syndrome coronavirus 2
ICHD-3: International Classification of Headache Disorders, 3rd edition
SD: Standard deviation
IQR: Interquartile range
CI: Confidence interval
ITT: Intention to treat
PP: Per protocol
FDR: False discovery rate
