# Supplementary material for: Frequency and phenotype of headache in covid-19: a study of 2194 patients
Source: Sci Rep. 2021 Jul 19;11:14674. doi: 10.1038/s41598-021-94220-6 (PMC8290038; doi:10.1038/s41598-021-94220-6)
Supplement: Supplementary file 1 — Supplementary Information. [file 41598_2021_94220_MOESM1_ESM.docx]

**FREQUENCY AND PHENOTYPE OF HEADACHE IN COVID-19: A STUDY OF 2194 PATIENTS**

**Supplementary materials:**

1. Supplementary figure 1: Screening of patients
2. Criteria for hospital admission.
3. Variable definitions.
4. List of the evaluated systemic symptoms.
5. List of evaluated red flags.
6. Prior history of headache.
7. Supplementary table 1: Frequency and type of general symptoms in the study sample.
8. List of acute medications used by the patients.
9. Expanded Authors list and contributions.

**Supplementary figure 1:** Screening of patients.

**
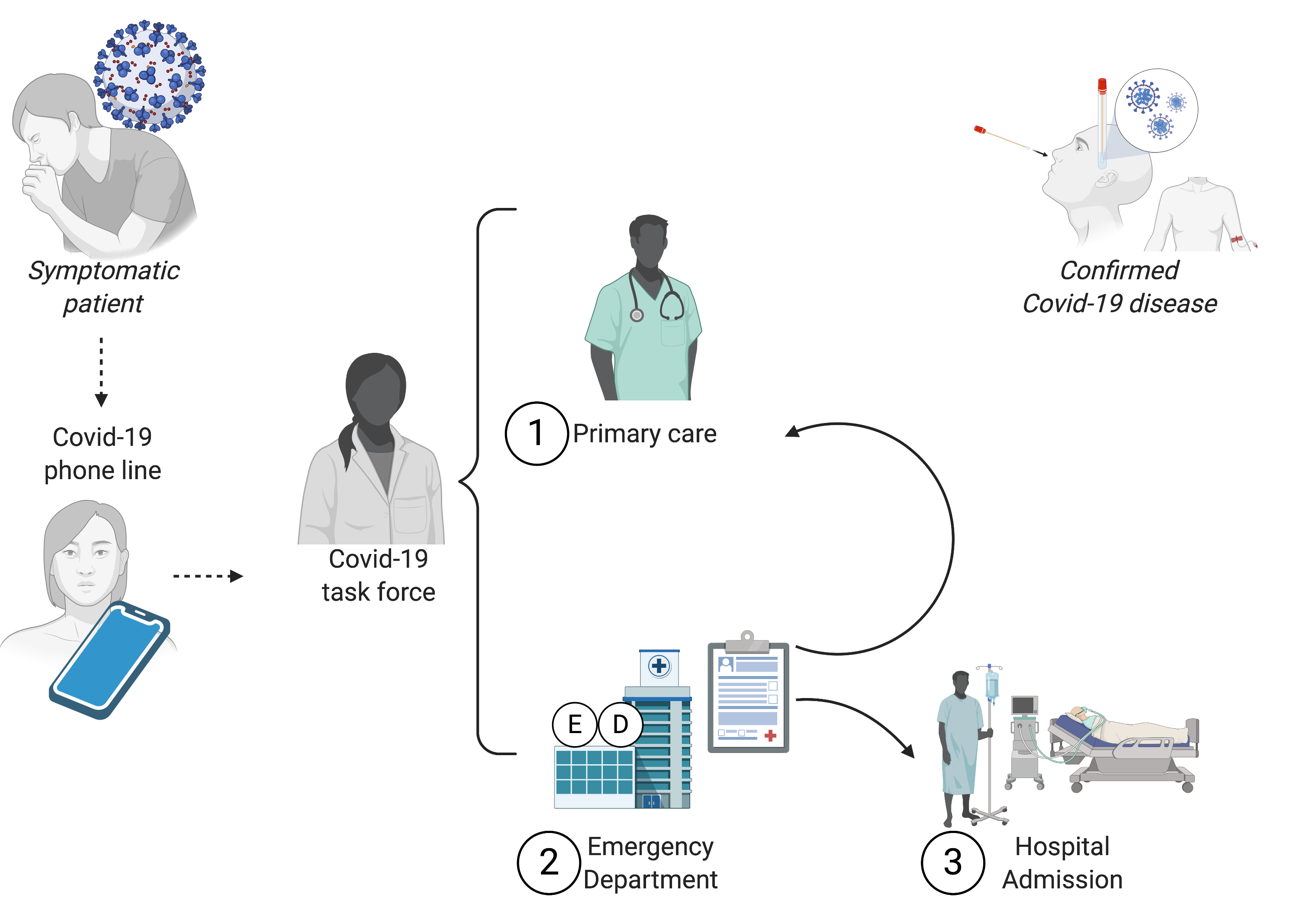
**

**Criteria for hospital admission:**

Presence of any of the following:

1) severe pneumonia (confirmed radiological diagnosis of pneumonia, either with X-ray and/or pulmonary computerized tomography, plus oxygen saturation <90% on room air or respiratory rate greater than or equal to 30 breaths per minute),

2) respiratory insufficiency (defined as partial pressure of oxygen in arterial blood sample less than or equal to 60 mmHg),

3) presence of poor prognostic factors in laboratory exams [increased levels of D-dimer (>500ng/dL), lactate dehydrogenase (>250 U/L), C-reactive protein (>5ng/L), lymphopenia (<900 cells x10^9^/L) or increased lactate (2mmol/L)]

4) age greater than 60 years and a prior medical history of select risk factors (e.g. pulmonary disorders, cardiovascular disorders, cancer, immunosuppression).

**Variable definitions:**

We analyzed variables according to the following definitions:

1. Arterial hypertension as: systemic blood pressure greater than 140/90 mmHg in two prior determinations.
2. Diabetes as: fasting blood glucose >126 mg/dl in two separate tests, HbA1c > 6.5%, blood glucose level >200 mg after oral glucose overload or blood glucose level >200 mg/dl with diabetes symptoms.
3. Smoking habit if patients were currently smoking or had discontinued in the preceding 6 months.
4. Cardiovascular diseases, including coronary artery disease, congenital heart diseases, cardiomyopathies, arrythmias, valvular heart disease, aortic aneurysms and peripheral artery disease.
5. Chronic pulmonary diseases, including chronic obstructive pulmonary disease (COPD), asthma, occupational lung diseases, interstitial lung diseases and pulmonary hypertension.
6. History of cancer, excluding cutaneous epidermoid and basal cell carcinoma.
7. Immunocompromised state, either congenital or acquired.
8. Prior history of neurological disorders. We used the WHO definition of disabling chronic neurological disorders (CND), as those neurological disorders that a) caused persistent disability, b) limited the individual’s functioning, and c) interfered with the person’s ability to engage in activities. We included conditions affecting both mental and physical function.

*Reference:*

Dua T, Janca A, Kale R, Montero F, Muscetta A, Peden M. “Public health principles and neurological disorders,”. In: World Health Organization: Neurological Disorders: public health challenges. ISBN 978-92-4-156336-9. World Health Organization (2006). P. 16-21.

**List of the evaluated systemic symptoms:**

Anosmia, asthenia, chest pain, cough, diarrhea, dyspnea, expectoration, fever, lightheadedness, myalgia, odynophagia, rash, syncope, weakness, or vomiting.

**List of evaluated red flags:**

Worst headache ever experienced, sudden onset of the headache (defined as abrupt onset that reaches the maximum intensity within five minutes and lasts for at least five minutes), wake-up headache (if the patient was woken up by the headache and not with the headache), treatment resistant headache (complete lack of response to acute medications), presence of altered mental status or confusion, loss of consciousness, and postural headache (precipitation or aggravation of the headache after sitting upright or standing, precipitation or aggravation of the headache by lying horizontally.

**Prior history of headache**

Prior history of headache was described by 223 (48.7%) patients, with migraine in 83 (18.1%), TTH in 99 (21.6%), other primary headache disorders in three (1.3%), one (0.4%) case of trigeminal neuralgia, and six (2.6%) cases of secondary headache. The remaining 31 (13.9%) did not have a formal headache diagnosis. The prior headache had been formally diagnosed by a primary care physician in 111 of those 223 (49.8%) cases, a neurologist in 35/223 (15.7%) cases and a headache specialist in nine (4.0%) cases. The remaining 68/223 (30.5%) patients with prior headache history had no formal headache consult.

**Supplementary table 1.**

Frequency and type of general symptoms in the study sample.

**Table 1.** Frequency and type of general symptoms in the study sample.

| **Symptom** | **Frequency (n, %)** | **Sample (n)** |
| --- | --- | --- |
| Asthenia | 337 (73.6%) | 458 |
| Cough | 294 (65.9%) | 446 |
| Anosmia | 269 (58.7%) | 458 |
| Fever | 263 (59.2%) | 444 |
| Weakness | 238 (53.4%) | 446 |
| Myalgia | 228 (51.5%) | 443 |
| Diarrhea | 185 (41.4%) | 447 |
| Dyspnea | 179 (40.3%) | 444 |
| Odynophagia | 127 (27.7%) | 458 |
| Lightheadedness | 100 (22.5%) | 444 |
| Chest pain | 99 (22.3%) | 444 |
| Expectoration | 65 (14.7%) | 443 |
| Rhinorrhea | 50 (11.2%) | 445 |
| Rash | 47 (10.3%) | 458 |
| Vomiting | 42 (9.5%) | 444 |
| Syncope | 10 (2.2%) | 458 |

**List of acute medications used by the patients:**

Symptomatic medication was needed by 413/437 (94.5%) patients, including acetaminophen in 382/413 (92.5%) cases, ibuprofen in 71/413 (17.2%) cases, and metamizole in 51/413 (12.3%) cases, dexketoprofen in 16/413 (3.9%) cases, triptans in 6/413 (1.4%) cases, tramadol in 5/413 (1.2%) cases, naproxen in 5/413 (1.2%) cases, aspirin in 4/413 (1.0%) cases, and etoricoxib in 2/413 (0.5%) cases.

**Expanded Authors list and contributions:**

| **Name** | **Location** | **Role** | **Contribution** |
| --- | --- | --- | --- |
| David García-Azorín, MD, MSci | Hospital Clínico Universitario de Valladolid | Author | Designed and conceptualized study; analyzed the data; drafted the manuscript for intellectual content |
| Álvaro Sierra, MSci | Hospital Clínico Universitario de Valladolid | Author | Collected data, conducted analysis, and drafted manuscript |
| Javier Trigo, MD | Hospital Clínico Universitario de Valladolid | Author | Collected data, conducted analysis, and drafted manuscript |
| Ana Alberdi, MD | Valladolid East Primary Care Basic Health Area | Author | Collected data, revised and approved the manuscript |
| María Blanco, MD | Valladolid East Primary Care Basic Health Area | Author | Collected data, revised and approved the manuscript |
| Ismael Calcerrada, MD | Valladolid East Primary Care Basic Health Area | Author | Collected data, revised and approved the manuscript |
| Ana Cornejo, MD | Valladolid East Primary Care Basic Health Area | Author | Collected data, revised and approved the manuscript |
| Miguel Cubero, MD | Valladolid East Primary Care Basic Health Area | Author | Collected data, revised and approved the manuscript |
| Ana Gil, MD | Valladolid East Primary Care Basic Health Area | Author | Collected data, revised and approved the manuscript |
| Cristina García, MD | Valladolid East Primary Care Basic Health Area | Author | Collected data, revised and approved the manuscript |
| Ana Guiomar Lozano, MD | Valladolid East Primary Care Basic Health Area | Author | Collected data, revised and approved the manuscript |
| Cristina Martínez Badillo, MD | Valladolid East Primary Care Basic Health Area | Author | Collected data, revised and approved the manuscript |
| Carol Montilla, MD | Valladolid East Primary Care Basic Health Area | Author | Collected data, revised and approved the manuscript |
| Marta Mora, MD | Valladolid East Primary Care Basic Health Area | Author | Collected data, revised and approved the manuscript |
| Gabriela Núñez, MD | Valladolid East Primary Care Basic Health Area | Author | Collected data, revised and approved the manuscript |
| Marina Paniagua, MD | Valladolid East Primary Care Basic Health Area | Author | Collected data, revised and approved the manuscript |
| Carolina Pérez, MD | Valladolid East Primary Care Basic Health Area | Author | Collected data, revised and approved the manuscript |
| María Rojas, MD | Valladolid East Primary Care Basic Health Area | Author | Collected data, revised and approved the manuscript |
| Marta Ruiz, MD | Valladolid East Primary Care Basic Health Area | Author | Collected data, revised and approved the manuscript |
| Leticia Sierra, MD | Valladolid East Primary Care Basic Health Area | Author | Drafted and revised manuscript, developed study concept/design |
| María Luisa Hurtado, MD | Valladolid East Primary Care Basic Health Area | Author | Drafted and revised manuscript, developed study concept/design |
| Ángel Guerrero-Peral, MD, PhD | Hospital Clínico Universitario de Valladolid | Author | Drafted and revised manuscript for intellectual content, developed study concept/design |
